# Hyponatraemic seizure in a healthy adult due to stress-associated non-osmotic vasopressin–mediated antidiuresis on a low-solute background: a case report

**DOI:** 10.1186/s12245-026-01137-w

**Published:** 2026-02-13

**Authors:** Ninh Xuan Nguyen, Thi Kim Thanh Vo, Huong Thi Thanh Le, Quoc Viet Tran, Hang Ngoc Thuy Tran, Ngoc Tien Pham

**Affiliations:** Emergency Department, Vinmec Central Park International Hospital, Vinmec Healthcare System, Ho Chi Minh City, Vietnam

**Keywords:** Hyponatraemia, Seizure, Inappropriate antidiuresis, Low-solute intake, Stress-related hyponatraemia, Autocorrection, Urine osmolality, Non-osmotic vasopressin

## Abstract

**Background:**

Hyponatraemia is the most common electrolyte disorder and a recognised precipitant of acute symptomatic seizures. In addition to drug-induced, endocrine, and central nervous system causes, acute psychological stress can provoke non-osmotic AVP secretion with inappropriate antidiuresis (SIAD-like physiology) and, when combined with low-solute intake (“beer-potomania” spectrum), impair free-water clearance. A critical challenge is autocorrection—a rapid, spontaneous rise in serum sodium once stress abates and solute intake resumes. While this may prevent recurrence, it also carries a high risk of osmotic demyelination syndrome (ODS) if unrecognised.

**Case presentation:**

A healthy 40-year-old Korean man experienced a generalised tonic–clonic seizure after approximately 10–12 h of police interrogation with fasting and marked stress; history suggested recent low-solute intake with episodic heavy alcohol use. On arrival he was euvolaemic with sodium 121.6 mmol/L, serum osmolality 262 mOsm/kg, urine osmolality 540 mOsm/kg, and urine sodium 48 mmol/L. Brain MRI and EEG were normal; an incidental zygomatic–temporal vascular malformation was non-causal. Hypertonic saline was considered but deferred given clinical improvement and anticipated autocorrection. He received isotonic saline (about 1 L over 24 h) with sodium monitored every 2–4 h. Levels rose to 133.3 mmol/L within 11 h and 135.4 mmol/L by 36 h, then stabilised. He required no antiseizure therapy and was discharged on day 2. At 1 month, sodium was 139.8 mmol/L with no ODS.

**Conclusion:**

This case illustrates a dual-hit mechanism- stress-associated non-osmotic arginine vasopressin secretion with inappropriate antidiuresis (SIAD-like physiology) on a low-solute background-leading to acute symptomatic hyponatraemia. It highlights autocorrection as a distinct physiological phenomenon that mandates strict monitoring and readiness to re-lower sodium to ensure safe outcomes.

## Introduction

Hyponatraemia is the most common electrolyte disorder and a recognised trigger of acute symptomatic seizures. In hypotonic states, non-osmotic arginine vasopressin (AVP) release reduces renal free-water excretion, promotes cerebral oedema, and lowers seizure threshold. Common causes include medications (e.g. thiazides, SSRIs), central nervous and pulmonary disease, and endocrine disorders. Guidelines for first-seizure evaluation emphasise identifying metabolic disturbance alongside neuroimaging and EEG, in practice including serum electrolytes [1–3].

Less recognised are non-organic precipitants in otherwise healthy adults: namely acute psychological stress provoking non-osmotic AVP secretion with inappropriate antidiuresis (a SIAD-like phenotype) [4, 5], compounded by low-solute intake (fasting or “beer-potomania”) [6, 7]. Together these can produce clinically significant hypotonic hyponatraemia and seizures. Clinical risks are twofold: recurrence if diagnosis is delayed, and osmotic demyelination from over-rapid correction, including spontaneous “autocorrection” once triggers abate. Contemporary guidance supports strict correction limits and, in selected patients, desmopressin-clamp strategies to control the sodium rise [1, 2, 8–10].

Aim: to describe a dual-mechanism case— stress-associated non-osmotic AVP–mediated antidiuresis on a low-solute background—with full osmolality profiling, therapeutic considerations to avoid over-correction, and learning points for first-seizure pathways.

## Case presentation

### Patient information

A previously healthy 40-year-old Korean man (height 177 cm, weight 61 kg, BMI 19.5 kg/m²) with no chronic medications and no prior seizures. He denied use of diuretics, SSRIs, antiepileptics, or other hyponatraemia-associated drugs.

### Clinical findings

After approximately 10–12 h of police interrogation with no food intake and marked psychological stress, history obtained at admission suggested very low dietary solute intake over the preceding week with episodic heavy alcohol use (beer-potomania spectrum). The patient reported no intentional excessive water drinking and no large-volume fluid ingestion immediately prior to detention; however, the exact volume and timing of fluid intake in the 24 h preceding presentation could not be reliably quantified. During the interrogation period, he reported negligible oral intake, with no sustained access to free water.

Index event: a witnessed generalised tonic–clonic seizure with loss of consciousness, eye-rolling, frothing, lip bite, and post-ictal drowsiness.

Arrival vitals (ED, 20:59, 26/06/2025): BP 192/115 mmHg, HR 101 bpm, RR 20 breaths/min, T 36.8 °C, SpO₂ 97% (room air).

Examination: drowsy but arousable; no meningeal signs, no focal deficits; clinically euvolaemic. No features of hypertensive emergency (no encephalopathy, retinal or renal injury); BP improved with rest. There were no clinical features suggestive of alcohol withdrawal at presentation (no tremor, diaphoresis, agitation or hallucinosis); the timing of last alcohol intake was not precisely established.

### Timeline

Key time points for symptom onset, investigations, neurophysiology, imaging, and sodium trajectory are summarised in Table 1 (Timeline).

**Table 1:** (Timeline): Onset, labs, imaging, EEG, correction trajectory

Date–Time	Event / Result
26/06 20:59	ED arrival after generalised seizure
26/06 22:08	Na⁺ 121.6 mmol/L; K⁺ 3.62; Cl⁻ 87.6
26–27/06	Brain MRI w/wo contrast: no acute intracranial lesion
27/06 06:59	CBC: WBC 11.0 × 10⁹/L (NE% 84.1)
27/06 08:00	Serum Osm 262 mOsm/kg; Urine Osm 540 mOsm/kg; Urine Na 48 mmol/L
27/06 08:54	Na⁺ 133.3 mmol/L
27/06	EEG: normal
28/06 09:09	Na⁺ 135.4 mmol/L
28/06 16:00	Discharge, asymptomatic
28/07	Follow-up: normal; Na⁺ 139.8 mmol/L; seizure-free

### Diagnostic assessment

Initial laboratories (22:08, 26/06): Na⁺ 121.6 mmol/L, Cl⁻ 87.6 mmol/L, K⁺ 3.62 mmol/L, creatinine 89.7 µmol/L (eGFR 92 mL/min/1.73 m²), WBC 15.6 × 10⁹/L (neutrophil-predominant), CRP 1.65 mg/L. High-sensitivity troponin I 6.2 ng/L (0.0062 ng/mL; assay upper reference limit not exceeded). Bedside capillary glucose was 6.4 mmol/l.

Neuroimaging/EEG: Brain MRI without contrast (late 26/06) and with contrast (morning 27/06) showed no haemorrhage, infarct, or venous thrombosis; an incidental left zygomatic–temporal soft-tissue vascular malformation was noted (non-causal). Routine EEG (27/06) was normal. ECG: sinus tachycardia 105 bpm.

Osmolality studies (08:00, 27/06): measured serum osmolality 262 mOsm/kg, urine osmolality (Uosm) 540 mOsm/kg, urine sodium (UNa) 48 mmol/L; TSH 2.10 mIU/L and AM cortisol 398 nmol/L were normal. Samples were obtained after limited fluids; interpretation considered potential dilutional effects. Urine indices were not obtained at ED presentation; urine osmolality and urine sodium were measured the following morning after limited intravenous fluids and therefore should be interpreted as supportive rather than diagnostic of the initial mechanism. The inappropriately high Uosm (urine osmolality) supported ongoing non-osmotic AVP activity. The full electrolyte and osmolality profile is presented in Table 2.

**Table 2:** Electrolyte & osmolality profile (serum/urine) with reference ranges

Analyte	Result (date–time)	Reference range*	Interpretation
Serum sodium (Na⁺)	121.6 mmol/L (26/06, 22:08)	135–145 mmol/L	Profound hyponatraemia (by concentration); severe symptoms at presentation
Serum sodium (Na⁺)	133.3 mmol/L (27/06, 08:54)	135–145 mmol/L	Rapid rise with therapy; spontaneous aquaresis likely
Serum sodium (Na⁺)	135.4 mmol/L (28/06, 09:09)	135–145 mmol/L	Back within reference range
Serum sodium (Na⁺)	139.8 mmol/L (28/07, 08:00)	135–145 mmol/L	Normal at 1-month follow-up
Serum osmolality (P-osm)	262 mOsm/kg (27/06, 08:00)	275–295 mOsm/kg (lab-dependent)	Low → true hypotonic state
Potassium (K⁺)	3.62 mmol/L (26/06, 22:08)	3.5–5.1 mmol/L	Low-normal
Chloride (Cl⁻)	87.6 mmol/L (26/06, 22:08)	98–107 mmol/L	Low
Creatinine	89.7 µmol/L (26/06, 22:08)	62–115 µmol/L (adult male)	Normal (eGFR approximately 92 mL/min/1.73 m²)
Urine osmolality (Uosm)	540 mOsm/kg (27/06, 08:00)	Context-dependent†	Inappropriately high for hypotonic hyponatraemia → active AVP (supports SIAD in euvolaemia)
Urine sodium (UNa)	48 mmol/L (27/06, 08:00)	Context-dependent‡	Consistent with SIAD in euvolaemia; interpret with diet/diuretic context

* Reference intervals may vary by laboratory

† Samples were obtained after limited fluids; interpretation accounted for possible dilutional effects. For mechanism: Uosm > 100 mOsm/kg indicates AVP activity; Uosm < 100 mOsm/kg suggests suppressed AVP (e.g. primary polydipsia/low-solute)

‡ UNa is diet/volume/diuretic-dependent; thresholds (approximately 30 mmol/L) are supportive, not definitive for SIAD in clinically euvolaemic patients

Urine osmolality was measured once, prior to the observed rapid rise in serum sodium. Serial urine osmolality and quantitative urine output during the subsequent correction phase were not available. Nevertheless, the brisk increase in serum sodium over a short interval, in the absence of hypertonic saline, is physiologically consistent with suppression of non-osmotic AVP release and the onset of aquaresis

### Interpretation and differential diagnosis

Overall, the findings were consistent with true hypotonic hyponatraemia in a clinically euvolaemic patient. Although urine indices were obtained the next morning and were not diagnostic of the admission state, the markedly elevated urine osmolality was consistent with ongoing non-osmotic AVP activity (SIAD-like antidiuresis). Taken together with the history of low-solute intake, the most plausible explanation was reduced osmole availability with superimposed stress-associated non-osmotic AVP secretion, which would limit free-water excretion and hinder spontaneous correction. Baseline serum sodium values prior to presentation were unavailable; therefore, the duration and degree of pre-existing hyponatraemia could not be established.

Differential diagnoses considered included:

- Primary epilepsy – considered unlikely given the absence of prior seizures, normal MRI and EEG, and clear provoked metabolic context.
- Central nervous system infection or structural lesion – not supported by clinical assessment or neuroimaging.
- Drug-induced hyponatraemia – excluded, as the patient reported no exposure to diuretics, SSRIs, antiepileptics, or other implicated medications.
- Endocrine causes – thyroid function was normal; findings were not suggestive of adrenal insufficiency, and dynamic testing was not clinically indicated.
- Primary/psychogenic polydipsia – unlikely: there was no history of excessive water intake and urine osmolality was markedly elevated (Uosm 540 mOsm/kg), which argues against suppressed AVP typical of primary polydipsia.
- Pure low-solute hyponatraemia (beer potomania spectrum) – historical dietary history was supportive, but the markedly elevated Uosm (540 mOsm/kg) argued against appropriate AVP suppression, pointing instead to a mixed mechanism.
- Alcohol withdrawal seizure – considered; however, there were no autonomic or neuropsychiatric features of withdrawal, no seizure recurrence, and a clear metabolic precipitant (profound hypotonic hyponatraemia) was present, making withdrawal an unlikely primary cause.

Although prior serum sodium measurements were unavailable, the dietary history and biochemical profile suggest that hypotonic hyponatraemia likely pre-dated the period of acute stress, which subsequently limited renal free-water excretion and prevented spontaneous autocorrection.

### Therapeutic interventions

Because the seizure had fully abated before ED evaluation, mental status was rapidly improving, and autocorrection was anticipated once stress resolved and solute was reintroduced, guideline-default 3% NaCl bolus was considered but deferred. Instead, the team pursued careful isotonic saline (0.9% NaCl) as a controlled exception, given abated symptoms and the expectation of spontaneous correction, with every 2–4 h serum sodium monitoring during the first 24 h and subsequent spacing as stable. Cumulative 0.9% NaCl administration was approximately 1 L/24 h, with additional crystalloids limited to maintenance rates. A pre-specified desmopressin and/or 5% dextrose (D5W) re-lower plan was in place to cap correction if targets were exceeded, though ultimately not required. No benzodiazepines were needed post-event, and no maintenance antiseizure medication was initiated, as the seizure was deemed provoked and metabolic in origin. Given the uncertain chronicity of hyponatraemia, a desmopressin-based re-lowering strategy was predefined should serum sodium correction exceed recommended thresholds.

### Follow-up and outcomes

Serum Na⁺ rose to 133.3 mmol/L at 08:54 on 27/06 and 135.4 mmol/L at 09:09 on 28/06 (an increase of approximately + 11.7 mmol/L within 11 h, then stabilised). Urine output was monitored (no oliguria); the brisk rise was consistent with autocorrection risk after triggers abated. There were no recurrent seizures. The patient was discharged asymptomatic at 16:00 on 28/06 with counselling on fluid/solute strategy, alcohol cessation, and stress-reduction. At 1-month review (28/07) he remained well and seizure-free with Na⁺ 139.8 mmol/L. There was no clinical evidence of osmotic demyelination syndrome during admission or at follow-up.

## Discussion

### Pathophysiology – dual-hit model

**Pre-existing susceptibility from low-solute intake** Renal free-water excretion is constrained by daily osmole delivery (urea, sodium, potassium). Prolonged low-solute intake—particularly in the setting of fasting and alcohol-heavy diets—reduces this capacity and predisposes to dilutional hyponatraemia even with modest fluid intake. This physiology, classically described in the fasting or “beer potomania” spectrum, is well established [6, 7]. Such a background allows hyponatraemia to develop insidiously prior to presentation and lowers the threshold for acute neurological manifestations.

**Stress physiology (non-osmotic AVP secretion)** Acute psychological stress activates hypothalamic pathways and triggers non-osmotic arginine vasopressin (AVP) release, reducing renal free-water excretion and maintaining inappropriately concentrated urine despite hypotonic plasma [4, 5]. Importantly, stress-mediated AVP release in the absence of excess free-water intake does not, by itself, generate hyponatraemia; rather, it prevents spontaneous correction and increases neurological vulnerability. Accordingly, we describe this as stress-associated non-osmotic AVP secretion causing inappropriate antidiuresis (SIAD-like physiology) rather than stress as an aetiology of SIAD. In this case, the dietary history and biochemical profile suggest that hypotonic hyponatraemia likely pre-existed the prolonged interrogation, while sustained stress-related AVP activity during detention impeded autocorrection and contributed to seizure manifestation. The elevated urine osmolality at presentation supports ongoing AVP activity in this context.

**Convergence in this patient** The occurrence of a generalized seizure at a serum sodium concentration slightly above 120 mmol/L suggests an acute or rapidly evolving process superimposed on an existing vulnerability. Such a presentation is more consistent with a relatively rapid decline in serum sodium or impaired physiological adaptation, rather than with slowly progressive chronic hyponatraemia alone. In pure low-solute hyponatraemia, urine osmolality is typically below 100 mOsm/kg due to appropriate AVP suppression. In contrast, this patient’s urine osmolality of 540 mOsm/kg indicated persistent non-osmotic AVP activity, consistent with stress-related inhibition of water excretion on a low-solute background. Although osmolality measurements were obtained after limited fluid administration, the magnitude of urine concentration strongly supports this dual-hit mechanism, explaining both the seizure presentation and the subsequent rapid spontaneous rise in serum sodium once stress abated and solute intake resumed [2, 11, 12].

### Diagnostic pearls

In this first-seizure presentation, early identification of a metabolic trigger was essential. Initial testing confirmed hypotonic hyponatraemia, and subsequent urine studies helped delineate the underlying mechanism. A urine osmolality greater than 100 mOsm/kg indicates ongoing arginine vasopressin (AVP) activity and argues against pure low-solute states or primary polydipsia, in which urine osmolality is typically below 100 mOsm/kg [2, 11, 12]. In euvolaemic SIAD-like states, urine sodium is commonly ≥ 30 mmol/L, whereas hypovolaemia more often presents with lower values, although diuretics may confound this distinction [1, 2].

In the present case, the combination of fasting with negligible fluid intake, urine osmolality of 540 mOsm/kg, and urine sodium of 48 mmol/L strongly excluded primary polydipsia and supported a mixed mechanism: reduced osmole availability combined with non-osmotic AVP activity [2, 4, 12]. Although urine indices were obtained after limited fluid administration, their magnitude remained consistent with persistent AVP-mediated water retention.

Additional supportive markers such as serum uric acid or fractional excretion of urate were not available; while helpful in some contexts, these indices are not universally reliable, particularly in thiazide-associated hyponatraemia [11–13]. Clinical response to isotonic saline was interpreted cautiously, as improvement suggests hypovolaemia but does not reliably exclude SIAD [1, 2].

Alcohol withdrawal was considered as an alternative seizure aetiology; however, the absence of autonomic or neuropsychiatric features and the presence of profound hypotonic hyponatraemia with inappropriately concentrated urine supported a metabolic mechanism related to stress-associated AVP activity on a low-solute background.

### Therapeutic nuance – avoiding osmotic demyelination syndrome (ODS)

**Correction limits and risk stratification** Current guidance recommends limiting serum sodium correction to ≤ 8–10 mmol/L within 24 h and ≤ 18 mmol/L within 48 h; in patients at higher risk (alcohol use disorder, malnutrition, liver disease, hypokalaemia, or suspected chronic hyponatraemia), many experts favour a stricter ceiling of ≤ 8 mmol/L per 24 h (some ≤ 6–8) [1, 2]. In our patient, serum sodium rose from 121.6 to 133.3 mmol/L (an increase of approximately 11.7 mmol/L over 11 h) before stabilising, consistent with spontaneous autocorrection following stress resolution and reintroduction of dietary solute. Physiologically, autocorrection reflects suppression of non-osmotic AVP release once acute triggers abate, combined with renewed osmole delivery, leading to abrupt aquaresis and a rapid rise in serum sodium despite modest isotonic fluid administration [4, 5, 14]. This underscores the need for frequent (every 2–4 h) sodium monitoring, strict fluid balance, and early recognition of accelerating correction trajectories [1, 14]. Although serial urine osmolality or urine volume measurements during this phase were unavailable, the rapid rise in serum sodium strongly suggests abrupt aquaresis following AVP suppression, a recognised hallmark of spontaneous autocorrection.

**Desmopressin clamp – proactive versus reactive strategies** Two conceptually distinct desmopressin strategies are described. A proactive desmopressin clamp entails early administration of desmopressin to fix urine osmolality and tightly control the rate of sodium correction from the outset, whereas a reactive desmopressin clamp is initiated only in response to an unexpectedly rapid rise in serum sodium or emerging aquaresis. Evidence comparing these approaches remains limited and heterogeneous; a small randomised trial showed no difference in overcorrection rates, although the proactive strategy achieved a more controlled sodium rise within safety limits [10]. Observational data are similarly mixed [1, 9, 10]. In the present case, desmopressin was not administered; instead, a reactive strategy was planned with predefined thresholds for intervention, reflecting anticipated autocorrection once stress resolved and solute intake resumed. In scenarios where urine osmolality is relatively fixed, such as established SIAD or during a proactive desmopressin clamp, sodium correction can be further individualised using predictive equations to guide hypertonic saline dosing, including the Voets Eqs [15, 16]. These models are designed to estimate the expected change in serum sodium under controlled aquaresis and may enable more precise correction in selected patients. Given the rapid clinical improvement, uncertainty regarding hyponatraemia chronicity, and the descriptive scope of this case report, such a protocolised proactive approach was not applied. In this case, desmopressin was not administered upfront because the seizure had fully resolved before emergency department evaluation, neurological status rapidly normalised, and spontaneous correction was anticipated following resolution of stress and reintroduction of dietary solute. Nevertheless, the treating team explicitly recognised the risk of overcorrection in the setting of uncertain chronicity and ongoing non-osmotic AVP suppression. A reactive desmopressin strategy was therefore planned, with predefined thresholds for intervention should serum sodium rise accelerate or exceed safety limits.

**Choice of saline and timing of intervention** For patients with ongoing severe neurological symptoms, hypertonic 3% saline boluses remain the guideline-recommended initial therapy [2]. When symptoms have abated and autocorrection is anticipated, a desmopressin-based strategy may be considered to guide a controlled, incremental rise in serum sodium. Isotonic saline should generally be avoided in pure SIAD unless hypovolaemia is present, in which case cautious repletion under close laboratory surveillance is appropriate [1, 2]. Although hypertonic saline followed by desmopressin is recommended for patients with ongoing severe neurological symptoms, this approach was deferred because the seizure had already terminated, mental status was improving, and there were no persistent features of severe cerebral oedema. In this context, immediate hypertonic therapy was judged more likely to exacerbate overcorrection risk than to provide additional neurological benefit. This individualized decision should not be generalised, and guideline-recommended hypertonic saline remains the standard of care for patients with active or refractory neurological manifestations.

**Re-lowering strategies** Criteria for re-lowering were predefined as a serum sodium increase exceeding 10 mmol/L within 24 h or evidence of accelerating aquaresis. In such circumstances, prompt re-lowering with 5% dextrose, with or without desmopressin, is recommended to return serum sodium to a safer range before resuming controlled correction [1, 9].

**Why ODS did not occur here** The absence of osmotic demyelination syndrome in this patient likely reflects a short duration of hyponatraemia, early recognition, and close subsequent monitoring. Nevertheless, multicentre data demonstrate that ODS can occur even without exceeding conventional correction thresholds, reinforcing the need for vigilance and readiness to intervene [8].

**Dynamic monitoring for aquaresis** Increasing urine output accompanied by a decline in urine osmolality represents an early marker of aquaresis and impending autocorrection and should prompt intensified monitoring and consideration of corrective strategies [1, 14].

### Incidental imaging

The left facial soft-tissue vascular malformation was extra-cranial, non-causal, and unrelated to hyponatraemia. It should be documented as an incidentaloma with non-urgent maxillofacial/vascular review for baseline assessment and follow-up.

### Study limitations

This case has several limitations. Osmolality studies were obtained the morning after admission, after limited fluid administration, which may have influenced absolute values; pre-fluid serial measurements were unavailable. The presence of stress-related non-osmotic AVP activity was inferred physiologically, as copeptin/AVP levels and supportive indices such as serum uric acid or fractional excretion of urate were not measured. Quantitative water–solute balance data (24-h urine volume, precise oral intake, serial body weights) were incomplete, limiting precise temporal separation between spontaneous autocorrection following stress resolution and the effects of supportive therapy. Management deviated from default hypertonic saline boluses because neurological symptoms had abated and autocorrection was anticipated; this patient-tailored approach may not be generalisable to patients with ongoing severe symptoms. Follow-up was limited to 1 month without delayed neuroimaging, so subclinical osmotic demyelination syndrome or late recurrence cannot be excluded. A contributory role of alcohol withdrawal cannot be definitively ruled out given uncertainty regarding the timing of last alcohol intake, although clinical features were not supportive.

### Clinical implications & future directions

This case underscores several implications for the evaluation and management of hyponatraemic seizures. Early serum and urine osmolality with urine sodium, obtained before substantial fluid administration when feasible, are central to distinguishing non-osmotic AVP–mediated states from low-solute or hypovolaemic mechanisms. In euvolaemic patients, a urine osmolality greater than 100 mOsm/kg supports ongoing AVP activity, even when dietary solute intake is reduced.

Safe correction hinges on strict correction ceilings, frequent early sodium monitoring, and awareness of aquaresis and spontaneous autocorrection once stress resolves and solute intake resumes. Hypertonic 3% saline remains the standard for patients with ongoing severe neurological symptoms, whereas desmopressin-based strategies and re-lowering with 5% dextrose may be considered to control or reverse overly rapid correction in selected cases. For seizures attributable to reversible metabolic disturbances, long-term antiseizure therapy is usually unnecessary once the underlying abnormality is corrected. Incidental extra-cranial findings should be managed separately to avoid diagnostic anchoring.

More broadly, stress-related hyponatraemia is likely under-recognised and may arise in diverse high-stress settings, including legal detention, military service, and occupational strain. Improved recognition of this mechanism and its correction dynamics may reduce preventable neurological morbidity.

Future studies should better characterise stress-associated euvolaemic hyponatraemia using biomarkers such as copeptin and serial osmolar indices, and define which patients benefit most from proactive versus reactive desmopressin strategies. Longer-term observational data are also needed to clarify neurocognitive outcomes and recurrence risk.

## Conclusion

Acute psychological stress on a low-solute background can precipitate symptomatic hypotonic hyponatraemia with seizure through non-osmotic AVP secretion and inappropriate antidiuresis (SIAD-like physiology). This case emphasises that early osmolality profiling is essential for clarifying the mechanism and guiding safe correction. Vigilant monitoring and strict correction limits are required to prevent over-rapid rises in sodium. Recognition of stress-related hyponatraemia has broad relevance in high-stress contexts worldwide, including legal, occupational, and military settings.

## Learning points

In first-seizure presentations, serum electrolytes and glucose should be obtained promptly, with early osmolality studies to define the mechanism of hyponatraemia.

A urine osmolality > 100 mOsm/kg in a euvolaemic patient suggests inappropriate AVP activity, even when low-solute intake is present.

Safe management depends on tight correction limits and frequent sodium monitoring, with readiness to re-lower if targets are exceeded.

Long-term antiseizure medication is usually unnecessary when the seizure is metabolic in origin; incidental findings should be documented but managed separately to avoid diagnostic anchoring.

## Data Availability

All relevant clinical data supporting the findings of this case are included within the article.
